# Satellite Remote Sensing of Harmful Algal Blooms (HABs) and a Potential Synthesized Framework

**DOI:** 10.3390/s120607778

**Published:** 2012-06-07

**Authors:** Li Shen, Huiping Xu, Xulin Guo

**Affiliations:** 1 State Key Laboratory of Marine Geology, Tongji University, Shanghai 200092, China; E-Mail: xuhuiping@tongji.edu.cn; 2 Department of Geography and Planning, University of Saskatchewan, Saskatoon, SK S7N 5C8, Canada; E-Mail: xulin.guo@usask.ca

**Keywords:** harmful algal blooms (HABs), satellite remote sensing, multiple-spectral sensors, hyperspectral sensor, spectra analysis, oceanographic parameters, a synthesized framework, multiple oceanographic explanations

## Abstract

Harmful algal blooms (HABs) are severe ecological disasters threatening aquatic systems throughout the World, which necessitate scientific efforts in detecting and monitoring them. Compared with traditional *in situ* point observations, satellite remote sensing is considered as a promising technique for studying HABs due to its advantages of large-scale, real-time, and long-term monitoring. The present review summarizes the suitability of current satellite data sources and different algorithms for detecting HABs. It also discusses the spatial scale issue of HABs. Based on the major problems identified from previous literature, including the unsystematic understanding of HABs, the insufficient incorporation of satellite remote sensing, and a lack of multiple oceanographic explanations of the mechanisms causing HABs, this review also attempts to provide a comprehensive understanding of the complicated mechanism of HABs impacted by multiple oceanographic factors. A potential synthesized framework can be established by combining multiple accessible satellite remote sensing approaches including visual interpretation, spectra analysis, parameters retrieval and spatial-temporal pattern analysis. This framework aims to lead to a systematic and comprehensive monitoring of HABs based on satellite remote sensing from multiple oceanographic perspectives.

## 1. Introduction

Harmful algal blooms (HABs) are deleterious phenomena characterized by the rapid accumulation of biomass in aquatic systems that have escalated worldwide in recent years. HABs have severe impacts on coastal ecosystems, fishery resources, and public health [1,2]. Three primary factors contribute to the occurrences of HABs: phytoplankton species, nutrition sources, and the dispersal mechanism. Eutrophication caused by anthropogenic activities has been determined to be one of the main sources of nutrition of HABs [3–6]. Furthermore, Anderson has pointed out that variation of oceanographic environmental parameters can also stimulate HAB events [7].

HABs can be generally classified into two categories: toxic and non-toxic [8]. The toxic species can directly release poisonous components causing paralytic shellfish poisoning (PSP), amnesic shellfish poisoning (ASP), neurotoxic shellfish poisoning (NSP) and diarrhetic shellfish poisoning (DSP). These toxic species only account for a few dozen of the thousands of known HAB species, but can cause severe diseases in human beings as well as aquaculture moralities [2]. Typical toxic species includes dinoflagellates (*Alexandrium* spp.), dinoflagellate (*Dinophysis* spp.) and diatoms (*Pseudo-nitzschia* spp.). The non-toxic species do not produce toxins, but can lead to aquaculture kills as a result of oxygen depletion or disturbance of the marine food web. That is why they are still called harmful algal blooms even though they produce no deadly toxins. Those phytoplankton are mainly known to include certain types of dinoflagellates (*Ceratium* spp., *Gymnodinium* spp.), diatoms (*Chaetoceros* spp., *Rhizosolenia* spp., *Prymnesiophyte* spp., *Phaeocystis* spp.) and ciliates (*Mesodinium* spp.), *etc*. Some of the aforementioned algal species can cause water discoloration when its abundance reaches a certain high level, which is usually referred to as red tides. Most red tide-forming species such as certain dinoflagellates (e.g., *Ceratium dens*, *Ceratium divaricatum*, *Gymnodinium sanguineum*, *Protoperidinium*), diatoms (e.g., *Rhizosolenia setigera*), prymnesiophyte flagellates (e.g., *Phaeocystis*) and ciliates (e.g., *Mesodinium rubrum*) are non-toxic [8], but some intensely toxic events of low species concentrations only dominating thin layers (subsurface blooms) do not cause the discoloration of water. Therefore, harmful algal bloom (HAB) is used as an obligatory term to encompass all the algal phenomena characterized by high biomass and/or toxin-production [9].

A growing number of global HABs have been reported at different international conferences, workshops and publications on this subject since 1974 [3,7]. Every year many coastal regions throughout the World are threatened by the serious ecological problems associated with huge economic losses and health issues caused by HABs. Hong Kong waters [10], East China Sea [3], Korean South Sea [11], Japanese Sea [12,13], the Gulf of Tokin [14], Arabian Sea [15], the coast of France, the coast of Portugal [16], New Zealand waters [17], the Galican Rias [18], Baltic Sea [19], the Gulf of Mexico [20], Washington [21], the Gulf of California [22], the coast of Florida [23], the Gulf of Maine [24], the coast of Nova Scotia [16], the coast of British Columbia [25] and the South African coast [26] are all areas subject to HABs with bewildering tendencies of larger spatial extents and higher frequencies. Therefore, both routine and emergency monitoring of HABs are necessary for those coastal areas, estuaries, bays and gulfs. Countries including the United States, Canada, Norway, Spain, Portugal, Ireland, China, Japan and Korea have invested a large amount of funds and efforts into HABs monitoring programs [11,18,27–30]. Therefore, the complex mechanism of HABs in the context of multiple oceanographic conditions requires a systematic understanding of the effects of different factors as well as their spatial-temporal patterns, which can help monitor and forecast HABs to reduce losses to the marine community [29].

HABs are marine phenomena characterized by large geographic and short temporal scales. Traditional efforts to identify HABs include *in situ* ship-surveys and laboratory analysis, but these have unavoidable limitations in time, cost, and labor which do not lend themselves to large scale monitoring over a short period [3,5]. As technology developed in 1970s, with the advantages of large-scale, real-time, and long-term monitoring, satellite remote sensing has been widely used to detect HABs as well as the oceanographic environmental characteristics that favor the formation of HABs [29]. Although it is difficult for satellite remote sensing to detect high toxicity HABs existing in thin layers, it still provides an effective tool for identifying high-biomass HABs such as red tides. However, current literature shows that the unsystematic understanding of HABs, the insufficient incorporation of satellite remote sensing, and a lack of multiple oceanographic explanations of HAB mechanisms are the major problems for remote sensing of HABs. A synthesized framework integrated with different remote sensing approaches is necessary to provide a systematical view and explanations of these complicated marine phenomena. In this study, we review the satellites sensors, techniques and algorithms for detecting HABs. Based on the challenges and opportunities found in existing remote sensing of HABs, a potential conceptual framework that combines all solvable strategies with multiple oceanographic explanations is proposed to provide a systematic way to detect HABs.

## 2. Satellite Remote Sensing of HABs

Compared to pure water, most HABs have distinct spectral characteristics (significant absorption bands in around 500 nm, 675 nm, and reflectance peaks in 550 nm and 700 nm) [31], which are caused by the dramatic increase of phytoplankton biomass. In particular, the chlorophyll fluorescence peak at 683 nm is a special characteristic of HABs which can be used to effectively separate it from other types of water. However, for some HABs the reflectance peak is shifted to 700 nm which is not caused by the fluorescence effect, but is contributed to by the elevated back scattering as a result of the increased phytoplankton density, or at least is a combination of the fluorescence and elastic scattering effects [32,33]. Different HAB species have distinct spectral characteristics. Zhao *et al.* concluded that three main different spectral characteristic types (the single-peak, the double-peak and the wide peak) exist for most HAB species. The single peak is characterized by a single reflectance peak at 680–750 nm (e.g., *Heterosigma akashiwo*, *Ceratium furan*) while the double-peak type has a strong reflectance peak at around 700 nm and a weak peak at around 800 nm (e.g., *Gymnodinium* spp., *Pyramimonas* spp.). The wide-peak type has a relatively broad reflectance peak distributed from 680 to 900 nm (e.g., *Platymonas spp., Nitzschia closterium* and *Chlorella* spp.) [34]. The aforementioned spectral responses are shown more obviously by intense HABs than in water with normal phytoplankton concentrations. These different characteristics can allow various satellite system with different spectral resolutions to detect different HABs by developing numerous algorithms.

### 2.1. Data Sources and Their Suitability for Monitoring HABs

#### 2.1.1. Multiple-Spectral Sensors

Since the first ocean remote sensing instrument, Coastal Zone Color Scanner (CZCS), was launched in 1978, a number of ocean remote sensing missions including Sea-viewing Wide Field-of-view Sensor (SeaWiFS), Moderate Resolution Imaging Spectrometer (MODIS), Medium Resolution Imaging Spectrometer (MERIS), Ocean Color Monitor(OCM) series and Hyperion, were developed to measure various marine biophysical and biochemical parameters (Table 1). These remote sensors supply a series of ocean color imagery which have been successfully applied in pigment concentration estimation and Sea Surface Temperature (SST) retrieval, playing a vital role in marine environmental management. The Advanced Very High Resolution Radiometer (AVHRR), a sensor carried on National Oceanic and Atmospheric Administration (NOAA) and mainly designed for climate change study, can also provide SST for HABs detection. Previous studies have shown great potential of these satellite data for remote sensing of HABs monitoring.

##### CZCS (1978–1986)

CZCS, operated from 1978 to 1986, has six spectral bands (443 nm, 520 nm, 550 nm, 670 nm, 750 nm and 11.5 μm), five of which are located in the characteristic region of HABs spectra, providing the first opportunity for satellite observation of HABs by quantifying phytoplankton pigment concentrations [20]. In 1978, CZCS was successfully utilized to detect *Karenia brevis* blooms by identifying a highly chlorophyll discolored anomaly in the Gulf of Mexico [35]. Several subsequent studies on monitoring HABs by CZCS were conducted by [23,36,37]. However, as [38] mentioned, due to the infrequency and short living period of HABs, CZCS could not be used to routinely monitor HABs because of its delays in data collecting and processing, which led to relatively little research being found in publications [20].

##### SeaWiFS (1997–2010)

As the second generation of ocean sensors, SeaWiFS was launched in SeaSTAR in 1997, which stopped collecting data in 2010. Compared with CZCS, SeaWiFS has more potential for initiating routine monitoring of chlorophyll concentration which is considered an effective means to identify HABs. The advantages of SeaWiFS can be demonstrated by the additional four spectral bands (412 nm specific to the absorption of yellow substances, 490 nm sensitive to the chlorophyll variation, 765 nm and 865 nm more suitable to atmospheric correction), and daily imagery can also enable the real-time detecting of variation in short-lived HABs [31]. SeaWiFS data has contributed significantly to global HAB monitoring in the past 15 years. SeaWiFS imagery integrated with other ancillary datasets played an important role in monitoring *Karenia brevis* blooms of the CoastWatch program in September 1999, Florida, initiated by the National Oceanic and Atmospheric Administration's (NOAA).

Such an effort allowed the distribution of “Harmful Algal Bloom Bulletins” nationwide for effective responses to HABs in the Gulf of Mexico [20]. The potential of SeaWiFS data has also been explored in the *G. catenatum* blooms in New Zealand waters [39], the South China Sea [14], the Pearl River Estuary [10], and the Baltic Sea [40] based on the abnormal chlorophyll concentration shown by the imagery. Unfortunately, SeaWiFS has no bands designed in the 683 nm region (the florescence peak of chlorophyll) which is a significant spectral indicator of HAB occurrence [33]. In addition, inappropriate atmospheric correction resulting from poor knowledge of aerosol conditions, absorption by colored dissolved organic matter (CDOM), scattering by inorganic suspended components, and reflection by a shallow bottom can all impact the optical properties detected from the imagery [40]. Additionally, lack of contemporaneous *in situ* validation data can restrict the accuracy of applying SeaWiFS in HAB detection [3]. Reinart and Kuster in particular have emphasized the limitations of SeaWiFS data in the detection of heavy algal blooms because of the high water-leaving radiance in near infrared regions [40]. The relatively coarse spatial resolution of SeaWiFS data limits the study area of HABs to a large spatial scale (>1,000 km^2^) which is often found in coastal areas such as the East China Sea, Bohai, the Gulf of Mexico and the Korean coastal waters [3,20,41].

##### MODIS (Terra/1999–Present and Aqua/2002–Present)

MODIS, a third generation sensor for oceanic satellite observations, was launched on both Terra and Aqua satellites in 1999 and 2002, respectively. It can provide daily imagery of 36 bands at three spatial resolutions (250 m for bands 1 and 2, 500 m for bands 3 through 7, and 1,000 m for bands 8 through 36). Especially bands 8 to16 in the 405–877 nm spectral region are specifically designed for studying ocean color, phytoplankton concentration as well as biogeochemistry [31]. Compared with SeaWiFS, the primary advantage of MODIS is that the particularly designed fluorescence band (676 nm) can be used to detect HABs based on a fluorescence line height (FLH) calculation for the coastal optically-complex water, the optical spectra of which is dominated by CDOM [34]. Such exploration has been conducted in the Gulf of Mexico and in the Bohai Sea, and the results showed a good correlation between the satellite-derived information and *in situ* measurement due to the little impacts of atmosphere and suspended sediments [33,42]. Tomlinson *et al.* applied MODIS FLH imagery in a *K. brevis* bloom in the Gulf of Mexico and found 71% of the blooms could be identified [43]. SST information can also be obtained at the same time as chlorophyll concentration is retrieved for the same HAB occurrence which greatly improves the accuracy of HAB detection by integrating analysis of multiple satellite information [44]. Furthermore, MODIS also provides the opportunity to estimate the primary production for algal bloom water because of the availability of all the necessary parameters including chlorophyll concentration, SST, daily photosynthetically active radiation, and daily diffusion attenuation coefficient derived from MODIS. The spatial resolution of MODIS data can guarantee the accuracy for HABs with an area more than 1,000 km^2^ [44]. However, MODIS imagery suffers severely from the sunglint problem. Because the sensor was designed for observations of atmosphere, land and ocean, it does not tilt toward the track to avoid the solar flare influence. Another limitation is that the fluorescence region (676 nm) in MODIS is a little farther from the actual chlorophyll peak (683 nm), especially when the chlorophyll concentration is higher [34].

##### MERIS (2002–Present)

MERIS, another popular third generation satellite sensor, was launched on the ENVISAT-1 satellite in 2002 by the European Space Agency (ESA). It has 15 spectral bands (350–1,040 nm) at 300 m spatial resolution covering all the regions for ocean studies. Compared with MODIS, the fluorescence bands (681 nm and 709 nm) are closer to the actual chlorophyll peak position, and are, therefore, more suitable for detecting HABs based on FLH methods. MERIS can extract 78% of fluorescence information while MODIS can extract a mere 57% [45,46]. Additionally, the 620 nm band is more sensitive to the suspended materials and the 900 nm band responds better to the water content in the atmosphere. All of these advantages can improve the accuracy of radiometric correction of satellite imagery for obtaining reliable information of HABs [47]. In addition, MERIS band 6 (620 nm) and band 7 (665 nm) are respectively more sensitive to the absorption region (603 nm) and reflectance peak (650 nm) of cyanobacterial blooms while MODIS does not have this characteristic spectral band [48]. The suitability of MERIS for cyanobacterial bloom identification from other types of HABs compared to MODIS has also be demonstrated by Kuster *et al.* and Koponen *et al.* [49]. MERIS was also found to have more reasonable band design than MODIS for identifying other HABs including *Dicrateria zhanjiangensis Hu*, *Pyramimonas* sp. and *Nitzschia closterium* species. Furthermore, the spatial resolution of MERIS is superior to that of SeaWiFS and MODIS, which allows for more accurate detection of HABs with an area of less than 1,000 km^2^ in comparatively small water areas such as lakes and rivers. The disadvantage of using MERIS data for HAB studiers is that since ENVISAT is a commercial satellite providing no free data to researchers, the availability of data is limited, which restricts the operational observation of HABs [47]. Many potential uncertainties about the future status of the MERIS instrument pose another threat to its data availability for use in HAB detection [29].

##### AVHRR (1978 to Present)

AVHRR was aimed to study the global climate and environmental change with high temporal resolution (daily) and moderate spatial resolution (1.1 km × 1.1 km). There are four bands in the first AVHRR carried on the TIROS satellite (1978) and AVHRR/2 was enhanced to five bands (0.6, 0.9, 3.5, 11 and 12 um, respectively) initially aboard NOAA-7 (1981) [31]. Due to the operational real-time capability and two visible bands sensitive to phytoplankton scattering in coastal turbid water, AVHRR data were greatly explored in studying large-area HABs as shown by the amount of published literature [20,25,50]. More frequently, AVHRR data are exploited for SST information retrieval due to the thermal bands allowing information extraction of water mass movement associated with HABs [10,20]. However, compared with MODIS and MERIS, fewer spectral channels and lower spatial resolution limit the operational monitoring of HABs, AVHRR can merely be used to detect large-scale HABs (more than 1 km^2^) and is incapable of discriminating specific phytoplankton species within the HABs [47].

##### OCM (IRS-P4)/OCM-2(Oceansat-2)

Ocean Color Monitor (OCM) was launched onboard the Indian Remote Sensing Satellite IRS-P4 (Oceansat) in 1999 and completed its mission in 2000. It was specifically designed for oceanic observation including chlorophyll distribution, phytoplankton blooms, suspended matter movement, and atmospheric aerosol identification. It had eight spectral channels (404–882 nm) providing imagery with a spatial resolution of 360 × 236 m every two days for the same study area [51]. OCM-2, a sensor with the same configuration as OCM, was carried by Oceansat-2 in 2009 as the following mission of Oceansat. OCM series imagery have the common characteristic spectral bands (414 nm, 440 nm, 510 nm, 556 nm, 668 nm) of HABs, so it can be applied to general HAB detection. Saragngi and Mohammed detected dinoflagellate algal blooms in the Kerala coastal and Calicut waters by exploring OCM imagery based on the OC2 empirical algorithm, showing good correlation with the *in situ* data [52]. Utilization of OCM-2 data for HAB detection is rarely found in the published literature, perhaps due to the relatively short period since its launch in 2009. Although the spatial resolution of OCM series imagery is superior to SeaWiFS and MODIS (bands 3 through 36), it is still incapable of accurate identification of HABs due to the lack of a specific fluorescence band for developing FLH methods.

#### 2.1.2. Hyperspectral Instruments

Hyperspectral instruments are deemed a promising tool for future harmful algal bloom detection due to their continuous spectrum which allows for more accurate quantification of phytoplankton characteristics [29]. Previous literature has shown the potential of both *in situ* spectroradiometers (e.g., ASD Fieldspec) and onboard sensors in identifying HABs. Lee and Carder used field collected hyperspectral remote sensing reflectance to derive absorption spectra of phytoplankton pigments with an accuracy of 78.6%, which provided great possibility of retrieving more pigments information besides chlorophyll concentration from multiple-spectral sensors [53]. In addition, Randolph *et al.* showed that *in situ* hyperspectral reflectance collected by ASD Fieldspec can effectively estimate chlorophyll concentration and phycocyanin absorption characteristics of cyanobacteria HABs [54].

The most frequently used hypespectral satellite sensors are Hyperion and the Compact High Resolution Imaging Spectrometer (CHRIS). As the first civilian hyperspectral imaging spectrometer initiated by NASA's New Millennium Program (NMP), Hyperion was launched on the Earth Observing 1 (EO-1) satellite in November 2000 [55]. Compared to the aforementioned discrete bands of multispectral satellite sensors, Hyperion can provide spectrally continuous data in 196 spectral bands (355–2,577 nm) with each 10 nm width band comprising the visible through shortwave infrared region. In addition, the spatial resolution of Hyperion (30 m) is as high as that of the Landsat Thematic Mapper (TM); however, the spectral resolution for the latter is far inferior to the former for HAB detection. Although Hyperion was designed specifically for land applications, the spectral channels can cover the entire region for water remote sensing as well [55]. As shown by [56], Hyperion has been used to monitor cyanobacterial blooms in the western part of the Gulf of Finland in 2002 by estimating chlorophyll concentration based on a bio-optical model. This study also indicated that the chlorophyll derived from multispectral sensors yields an underestimated value due to the limitation of their spatial resolutions. In spite of its potential designed for coastal water monitoring, Hyperion still suffers from several shortcomings. The relatively longer revisit period (16 days) as well as the small coverage (7.7 × 185 km) do not allow for routine real-time HAB monitoring. Besides, the poor signal to noise ratio also restricts its wide application in HAB communities. However, Hyperion data can still serve as important ancillary data for HAB detection by other superior instruments [40]. CHRIS, onboard the Project for Onboard Autonomy (Proba) satellite launched by the ESA in October 2001, is capable of acquiring both hyperspectral and multi-angular data at a spatial resolution of 18 m in a wavelength range of 415–1,050 nm with a revisit period of seven days. Simultaneous observation in 19 bands out of the total 62 bands can provide environmental information for both land and coastal monitoring [57]. The potential of CHRIS in retrieving chlorophyll concentration and estimating phytoplankton biomass has been proven by [58] in monitoring cyanobaterial blooms based on an empirical model.

Despite the fact that airborne hyperspectral instruments with higher spatial and spectral resolutions can improve HAB detection, they are still far from offering routine and real-time monitoring due to the expensive flight costs and the limits of the geographical scope that the sensor can cover. Therefore, airborne hyperspectral instruments such as Airborne Imaging Spectrometer for Applications (AISA) and Push-broom hyperspectral imager (PHI) are more frequently used to validate satellite-derived information or detect HABs in small area such as bays, lakes or along the shore [49].

### 2.2. Available Remote Sensing Techniques for Monitoring HABs

The principle remote sensing techniques for detecting HABs are interpretation of discoloration, spectral analysis and oceanographic parameters retrieval.

#### 2.2.1. Interpretation of Discoloration

True-color and false-color satellite imagery generated by combining different spectral layers can be used to identify the presence of water discoloration caused by HABs [59,60]. True-color composite imagery has more advantages for visual interpretation since it can reflect the actual color of algal blooms, which allows for identification of specific phytoplankton species directly based on some empirical knowledge of the species. This has been successfully proven by previous studies on remote sensing of HAB to detect *Skeletonema costatums* [44,61]. In addition, harmful algal blooms caused by other phytoplankton species including coccolithophores, trichodesmium and cyanobacteria can also been effectively identified by observing the discoloration of waters [19,62–64]. However, it is difficult to obtain quantitative information for a HAB merely based merely on the observation of discoloration. Besides, not all HABs produce water discoloration since some color anomalies are caused by other materials such as sediment or CDOM. Therefore, examining discoloration is not totally reliable, particularly when the study area is located in an optically complex coastal area without sufficient field data for validation, but the discoloration method can still provide some general information about a potential bloom such as the location and extent of the event [60].

#### 2.2.2. Optical HAB Algorithms

Based on the premise that a unique spectral characteristic corresponds to a specific harmful bloom, remote sensing optical approaches for detecting HABs can be categorized into two major types. One is aimed at exploring the optical properties (absorption, backscattering and reflectance) of each component (CDOM, suspended sediment, water and chlorophyll) present in the HAB water to establish equations which can indicate the reliable relationship between the optical characteristics of each component and the total sensor signals [65–67] as follows:

$$\text{R}(\lambda )\approx {\text{b}}_{\text{b}}(\lambda )/(\text{a}(\lambda )+{\text{b}}_{\text{b}}(\lambda ))$$

where *R*, 𝞴, a(𝞴), and b_b_(𝞴) refer to the irradiance reflectance of water, the spectral band, the total absorption and the total backscatter at spectral λ respectively. *a* and *b_b_* are represented by:

$$\text{a}={\text{a}}_{\text{w}}+{\text{a}}_{\text{c}}+{\text{a}}_{\text{d}}+{\text{a}}_{\text{p}},\text{and b}={\text{b}}_{\text{bw}}+{\text{b}}_{\text{bs}}+{\text{b}}_{\text{bp}}$$

where w, c, d, p, s, are water, CDOM, detritus, phytoplankton and suspended sediment, respectively [68]. This relationship can be obtained generally by developing empirical, semi-analytical or radiation transfer models. However, as Stumpf and Tomlinson indicated, the performance of those algorithms is determined by the stability of each component's spectral characteristics [29]. For this optical method, many *in situ* data (both the spectral characteristics and component percentage) are required for the equation establishment and validation. This method is only feasible for investigating HABs in Case 1 waters (deep ocean with chlorophyll pigments as the dominant component, rarely influenced by organic and inorganic components) but not applicable in turbid coastal waters (Case 2 water) because of the complex optical signals contributed by CDOM and particulate inorganic materials (POM). Also, a significant error may be yielded if the accuracy of the atmospheric correction of the imagery cannot be guaranteed, which indicates that such satellite data without good radiometric correction is not suitable for HAB detection using this method [3].

Numerous spectral band algorithms have been developed to overcome the limitation of the standard optical algorithms for HABs detection. Those algorithms include the single band model, two bands difference/ratio model, and multiple bands difference/ratio model [47]. The single band method has been exploited for detecting coccolithophore HABs in the northeast coast of the Atlantic by [69] who set a threshold for the reflectance of the AVHRR first band (580–680 nm). When the threshold is reached, there is a potential risk of a coccolithophore bloom. The same method based on AVHRR data applied in the Baltic Sea by Kahru *et al.* also showed effectiveness in identifying the scope and frequency of nodularia blooms [19]. For MODIS data, Kuster *et al.* demonstrated that band 1 (620–670 nm) and band 2 (841–876 nm) are sensitive to the variation of cyanobacterial blooms. This was further supported by Duan *et al.* who found that a threshold of 0.1 for MODIS band 2 reflectance could be used as the indicator of cyanobacterial blooms [48,70]. The two bands difference/ratio model, the earliest of which for CZCS was developed by [36], who suggested that a threshold for the ratio of MODIS band 1 and band 2 had significant effectiveness in coccolithophore bloom detection. Also Stumpf and Tyler demonstrated that a threshold for the ratio of AVHRR band 1 and band 2 is capable of identifying HABs on the west coast of Canada when the chlorophyll exceeds 5 mg/m^3^ [50]. By taking the idea of Normalized Difference Vegetation Index (NDVI) in vegetation remote sensing, a NDVI algorithm was developed for HAB detecting by utilizing the reflectance of AVHRR band 1 and band 2 [71]. Multiple bands difference/ratio algorithms are more established for SeaWiFS data. Mao and Huang established a model C = (R(band1) − R(band3))/(R(band5) − R(band3)) for detecting gymnodinium HABs in the East China Sea while Gu *et al.* developed C = (R(band5) − R(band4))/(R(band4) − R(band3)) for detecting *Skeletonema costatum* HABs [72,73]. To overcome the inefficiency of optical models in coastal turbid waters, Ahn *et al.* utilized three water-leaving radiances (Lw) at 443 nm, 510 nm and 555 nm of *in situ* measurements to establish a red tide index RI = (Lw(510)/Lw(555) − Lw(443))/(Lw(510)/Lw(555) + Lw(443)), and then related the RI with HABs absorption characteristics to yield different thresholds for HABs (higher value) and turbid non-HAB water (low value) [3]. Compared with optical models, the above mentioned spectral bands calculation algorithms can show better capability in characterizing certain specific HABs in different waters with improved agreement with the *in situ* data. Such approaches can serve as useful tools in monitoring HABs in specific regions or regions of high similarities (e.g., water components, physical environments, and HAB species). However, they cannot be applied widely from region to region where huge difference exists in terms of water types and phytoplankton species. Thus far, it is still difficult to find a general single method to effectively detect HABs caused by distinct dominant species occurring in different water areas, which requires integrating multiple techniques for conducting comprehensive analysis to improve the accuracy of HAB detection.

#### 2.2.3. Oceanographic Parameters Retrieval and Analysis

Oceanographic parameters such as chlorophyll a (Chl-a) and SST derived from satellite data normally serve as effective indicators for HABs. Chl-a concentration, the most important property of the marine ecosystem, has a close correspondence with the concentration of HAB species. Several standard remote sensing algorithms for different satellite sensors have been developed to operationally estimate the chlorophyll concentration [74,75]. For SeaWiFS data, bands 490 nm and 555 nm have been utilized to establish the chl_oc2 algorithm based on an empirical relationship between the chlorophyll concentration and the selected irradiance reflectance. Chl_oc2 is suitable for chlorophyll estimate in Case 1 water. To improve the atmospheric correction of the chl_oc2 algorithm, another two bands (443 nm, 510 nm) are incorporated to develop a new maximum band ratio formulation called chlorophyll 4 algorithm (OC4). Although OC4 can maintain the sensor signal in the highest level, it still cannot yield accurate results in coastal turbid waters (Case 2 water) [75]. To solve this problem, MODIS Chlor_2 and Chlor_3 algorithms were developed by incorporating band 448 nm and are more suitable to estimate chlorophyll information from both Case 1 and Case 2 water [31]. SST imagery obtained by thermal infrared remote sensing technologies can provide a synoptic view of upwelling patterns, wind shifts and cooler waters from the pycnocline to reveal physical oceanographic process of HABs [15,30,76]. Both Chl-a and SST have been considered as indicators for studying HABs over large geographic scales for long-term monitoring [41]. Tang *et al.* investigated the short-term variability of phytoplankton blooms in the Arabian Sea by analyzing Chl-a derived from both Ocean Color and Temperature Scanner (OCTS) and SeaWiFS as well as SST derived from AVHRR [15]. By examining distributions of Chl-a and SST imagery, HAB events were also monitored in the Gulf Tonkin, Pearl River Estuary, the South China Sea, Bohai, the Gulf of Mexico, Vietnam waters, and New Zealand waters [5,10,14,15,17,20,37,41,77]. Nevertheless, those two parameters are insufficient in some cases. When blooms are caused by two or more types of phytoplankton, it is difficult to distinguish the toxic species producing only a small percentage of the total chlorophyll from a non-toxic one. Keafer and Anderson concluded that when HABs such as *Alexandrium* spp. do not dominate within the biomass blooms and then it was difficult to observe a bloom from the imagery using those estimated parameters [78]. Under these circumstances, *in situ* information, ecological associations, oceanographic and meteorological data are all required to supplement the detection of a potential HAB [10].

## 3. Challenges of Remote Sensing of HABs

With the increasing application of remote sensing in monitoring the mechanisms of HAB dynamics, advantages of remote sensing have improved the understanding of HABs within the whole field. However, by tracking a number of previous relative studies, we also found some issues existing in the remote sensing of HABs. Here, we focus on three significant aspects of current limitations: (1) an unsystematic understanding of HABs; (2) insufficient utilization of remote sensing; and (3) multiple explanations of HABs' mechanism.

### 3.1. Unsystematic Understanding of HABs

HABs are complex marine phenomena with complicated mechanisms involving a source of causative algal species, favorable oceanographic conditions, extra contributing factors and dispersal mechanisms [7]. Similarly, Chen *et al.* indicated that the formation of a HAB results from an intricate interaction of biological, chemical, physical and geological processes with associated contributors [79]. Stumpf and Tomlinson concluded that the linkage of ecological and physical forces with remote sensing technology would improve future HABs studies most effectively [60]. Therefore, a comprehensive and systematical understanding of the relationship between those multiple factors and HABs mechanisms is of great significance. However, to date few studies concerning the comprehensive pattern of HABs have been conducted especially in remote sensing of HABs. Most studies endeavor to explore the HAB mechanism from one or several aspects. For example, some primarily focused on the analysis of chlorophyll variation using empirical or bio-optical approaches in different coastal areas. Some combined the chlorophyll and SST analysis to predict conditions favorable for harmful algal blooms using ocean color imagery and meteorological data. Some studies focus on the chlorophyll and primary production characteristics of HABs. Other studies have developed HAB indices or classification algorithms based on the spectral characteristics of HAB waters. Microwave remote sensing derived sea wind data has also been incorporated into the optical remote sensing techniques by some studies to improve HAB detection, but still a synthesized remote sensing framework of HABs considering every possible factor is necessary for the breakthrough in approaching the true mechanism of HABs. Table A1 in Appendix shows such information with regards to some studies found in previous literature.

**Figure 1.**
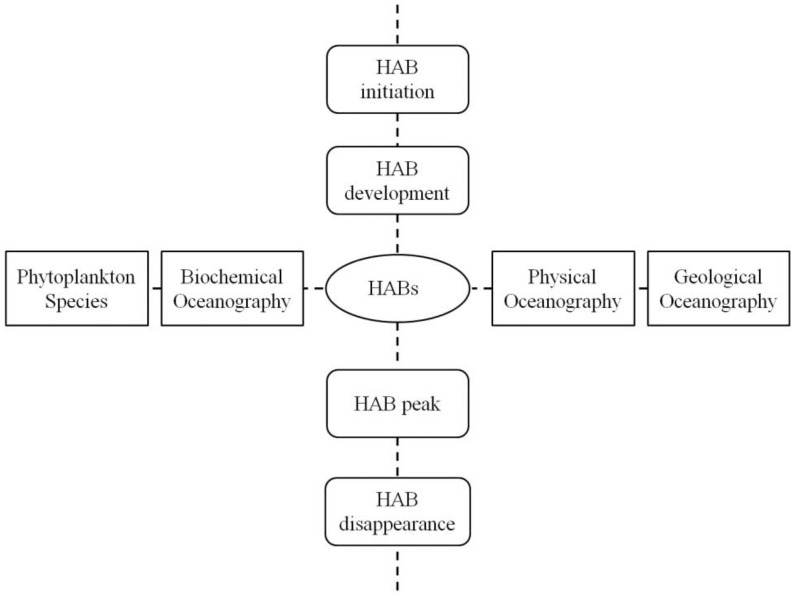
The cross pattern of HAB dynamic mechanism.

To help establish such a synthesized framework, we present a cross pattern of HABs mechanism (Figure 1) to aid in capturing the overall nature of HABs as much as possible. In this cross pattern, the Y axis shows temporal variations in the stages of initiation, development, peak, and disappearance of HABs. Stages closely connected to each other determine the total time a HAB can last. According to the information indicated by this arm, more attention should be given to approaches concerning the time scale of HAB problems and temporal resolutions of potential satellite data. By doing this more critical information can be captured to improve the accuracy of HAB detection.

The X axis integrates all the prerequisites and impacting factors of HABs indicating how all the horizontal aspects can reinforce and influence each other to induce a HAB. Zeng *et al.* concluded that phytoplankton species is the internal cause of HABs, and the biochemical factors are material base for HABs [80]. Physical process serves as the inducing force and geological oceanography provides the environment for HABs. This cross pattern links every aspect of HABs into one mode and presents a systematical view of the complex mechanism of HABs, while avoiding separation of HABs into different individual parts for research. To accomplish the comprehensive study of HAB mechanisms, remote sensing has great capabilities in exploring both the vertical and horizontal aspects shown in this cross pattern. A synthesized framework of remote sensing of HABs can be established to correspond to the cross pattern of HAB dynamic mechanism.

### 3.2. Insufficient Incorporation of Remote Sensing

Substantive studies indicate that optical remote sensing, particularly of ocean color, is of primary interest within the remote sensing of HAB communities. The citation database *Web of Science* shows that, as of 13 July 2011, 575 articles on the subject of remote sensing of HABs are available. Of these, 338 articles refer to chlorophyll (around 60% of the total), 130 articles focus on SST (23% of the total), and 13 are for microwave remote sensing. Ocean color parameters can provide an effective interpretation of HABs only when discoloration is present. Even obvious anomalies can be identified from ocean color imagery; however, they cannot fully reveal the driving force or physical mechanism of HAB formation. SST can reveal the circulation patterns of HAB waters, but not enough to cover all the physical reasons for HAB dynamics. Due to the complex mechanism of HABs, there are many possible parameters derived from multiple data sources that can provide additional information on HABs. Most researchers applied no more than three parameters to detect the oceanographic conditions of HAB dynamics. Therefore, it is necessary and promising to combine the optical and microwave remotely sensed data together to generate more than three oceanographic parameters for a HAB event, making the analysis more reliable and the results more informative. For example, exploration of microwave information such as sea wind data is an intriguing opportunity for supplementing the physical explanation of HABs. Net Primary Production (NPP) estimated by ocean color technology is another potential parameter to reveal the bio-chemical oceanographic conditions of HABs due to a close relationship between primary productivity and blooms in which HAB phytoplankton dominates most of the productivity [17,81]. However, a few studies of NPP are conducted in remote sensing of HABs; in the *Web of Science* database there are only 13 relative articles available among the total 575 articles.

Another major problem lies in the selection of remote sensing imagery of appropriate spatial, temporal and spectral resolution for HABs study. Although different sensors have different advantages in detecting HABs, the accurate detection of HABs, especially for coastal water, cannot still be achieved due to a lack of availability of simultaneously high spatial, high temporal and high spectral resolutions. Normally an algal bloom varies dramatically with rapid occurrence and rapid disappearance during several days [82]. High frequency (less than three hours) of observation is required to track the highly dynamic HABs impacted by multiple oceanographic phenomena such as diurnal winds, river runoff and upwelling currents [83]. Even the satellite imagery with daily revisit times (SeaWiFS, AVHRR, and MODIS) is hardly capable of monitoring real-time HABs; it is more difficult for lower temporal resolution imagery such as MERIS with a revisit time of 2–3 days to allow this application. Less than three images a week may restrict the efficiency of detecting the rapidly evolving HABs, and it is impossible for monthly-average or weekly-average data to capture the high spatial and temporal variability of a HAB. Therefore, continuous images of high revisit time (at least daily) are required to guarantee the accuracy of tracing highly dynamic HABs. However, the availability of sequential imagery is also dependent upon cloud conditions. In some circumstances, compromising strategies are needed for the choice of satellite data to fulfill different objectives. For studying multiple oceanographic conditions during short-term HABs, MODIS is a possible data source compared with other sensors due to the accessibility of its information which can be obtained from both Aqua and Terra satellites during one day [84]. More possible data choices can promote the acquisition of good-quality imagery with limited cloud contamination [85].

The spatial resolution of current satellite sensors also limit the application of remote sensing in HAB detection [3,10,15,20,41]. Imagery of low spatial resolution (less than 1 km^2^) such as SeaWiFS, AVHRR and MODIS can only effectively identify HABs with a dominated area more thousands of square kilometers, normally not working well in bays, fjords, estuaries, lakes and rivers. Even the 500 m MODIS, 300 m MERIS, or OCM imagery are still inaccurate for detecting HABs at a small spatial scale of less than 100 m^2^. Because sometimes even in a small geographic scope probably less than 30 m^2^ the chlorophyll can still vary significantly to cause a HAB event. Kuster pointed out that even Hyperion with 30 m spatial resolution still has the possibility of missing the spatial features of some cyanobacterial blooms [56].

Spectral resolution is another major concern for remote sensing of HABs. It is particularly difficult to separate the HABs caused by one dominant phytoplankton species from the HABs triggered by another species. MODIS, a relatively newly launched sensor which can provide data of 36 bands twice a day, have great advantages in generating multiple oceanographic parameters. This can guarantee the compatibility of different parameters in the same processing mode. However, MODIS does not have ocean color bands positioned in 630 nm and 659 nm responsive to the phycocyanin absorption and reflectance. The 648 nm spectral channel of MODIS is only sensitive to chlorophyll concentrations higher than 60 mg m^−3^ [40]. Davis indicated that the 709 nm band uniquely designed in MERIS can help identify HABs by accurately quantifying chlorophyll concentrations caused by increased phytoplankton biomass [83]. It has also been proved by [40] that an obvious spectral characteristic of band 709 nm can be used to detect some HABs (e.g., *Cyanobacteria* spp.) with a chlorophyll concentration higher than 10 mg m^−3^. MODIS is inferior to MERIS because of its lacking of such a near infrared band highly sensitive to chlorophyll variation. MODIS and MERIS both have spectral bands 676 nm (MODIS), 681 nm (MERIS), and 709 nm (MERIS) sensitive to the fluorescence peak (683 nm), which can effectively improve HAB detection. Although seldom remote sensors have the exact spectral channels necessary to capture the specific spectral characteristics of different HAB events, Reinart and Kuster argued that thus far such sensors are still the best instruments for routine and real-time detection of HABs [47].

### 3.3. Lacking Multiple Explanations of HABs Mechanism

Although satellite images of both ocean color and estimated parameters can demonstrate the location, extent, spatial and temporal variation of HAB events, they are still insufficient. To enhance our knowledge of the possible mechanism of HABs, an explanation from multiple perspectives involving biochemistry, physics, and geology needs to be proposed; one that takes overall oceanographic environmental conditions into consideration [15,44]. Stumpf and Tomlinson also emphasized that a promising direction of remote sensing of HABs is to link ecology together with physical processes [60]. However, the problem within current HAB studies is that potential HABs are commonly delineated through analyzing oceanographic parameters derived from satellite data but lack reasonable discussions of the distribution and dynamics of HABs. Why do HAB events occur in this area or why does this region suffer from HABs? Those critical but poorly known details pose limitations that block us from understanding the studied HAB event in a systematical sense. Major questions concerning the multiple explanations are: (1) What is the causative species of the HAB phytoplankton? And what are the characteristics of such species? (2) What is the nutrition source, from river discharge or from the subsurface layers by thermocline convection? (3) How does the physical oceanographic process work? (4) What are the geological characteristics (geomorphology and deposition) of the study area? (5) What environment impacts do these factors impose on HAB formation and development? As a HAB event results from systematical interactions of various factors in the complicated marine environment, a well-grounded interpretation of multiple oceanographic conditions of HABs is required from the analysis obtained by remote sensing and other accessible information.

## 4. A Synthesized Framework of Remote Sensing for Monitoring HABs

**Figure 2.**
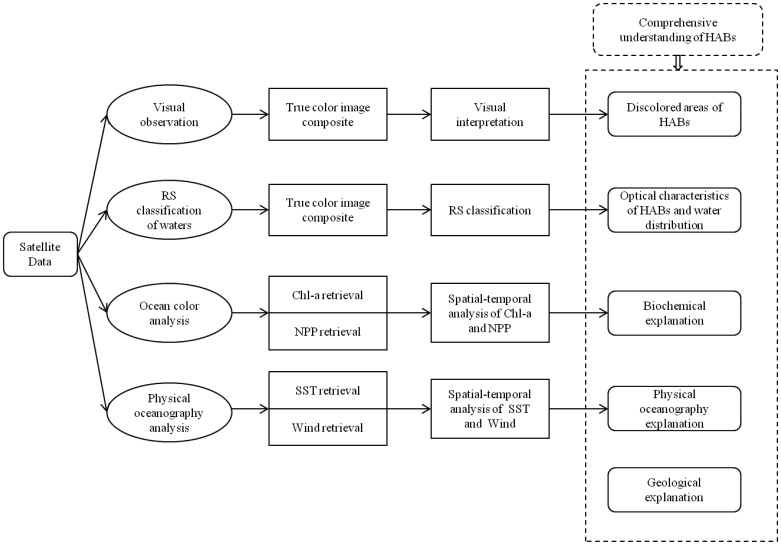
A synthesized framework of satellite remote sensing for detecting HABs.

Cullen *et al.* suggested that a system to synoptically understand the distribution and physiological information of phytoplankton in a complex oceanographic context is a critical application of HAB detection [86]. Anderson also highlighted the importance of multidisciplinary studies with large-scale physical and biological strategies in monitoring HABs with complex mechanisms [2]. Therefore, effective methods of integrating physiological ecology and modern oceanographic technologies are needed to increase the probability of tackling the aforementioned problems. Remote sensing has many different applications in HABs, but no single method is sufficiently suited to provide a synoptic view of HABs. By pointing out the division between HAB community and remote sensing community, Stumpf and Tomlinson [60] mentioned that the former have paid more attention to the associations of oceanography and ecology while the latter focus more on optical techniques. A coupling of ecology, physics and the appropriate implementation of remote sensing techniques can offer the greatest promise in HABs detection and forecasting [81]. A range of disciplines including biochemistry, physical oceanography and geology can be brought together to improve the identification of HABs. Therefore, we reviewed the recent literature regarding different remote sensing techniques, and combined those techniques with biochemical, physical oceanographic and geological strategies of HAB research. Finally, a synthesized framework of above mentioned knowledge transfer, optical and microwave techniques, and spatial-temporal analysis of HABs is proposed in this study, hopefully leading to a systematical understanding of HABs (Figure 2).

In this framework, four main components based on qualitative and quantitative remote sensing approaches can contribute to the comprehensive study of HABs. The primary approaches involve visual interpretation, classification, parameters retrieval, and image analysis which are recognized as key components of remote sensing strategies [87]. A variety of information concerning HABs is accessible such as the extent and location of the potential HAB, the distribution pattern of different types of waters, the spatial-temporal variation of marine environmental parameters of a HAB event, and multiple explanations for the HAB mechanism. This framework also has good potential to provide synoptic insight of HAB's dynamic processes (formation, development, peak, and disappearance) associated with important environmental factors such as nutrition and currents, which is critical in oceanography research of HABs [86]. A detailed illustration of this framework follows.

### 4.1. Visual Interpretation

Visual interpretation is the first step of optical processes within the synthesized framework. Generally, HABs can cause discoloration of the water, indicating a potential risk of a bloom. Some HABs are obviously recognized from either the true color or false color composite satellite images, so imagery interpretation can help with the identification of suspecting HABs for reporting, field data collection and further analysis. For easily detected species such as cyanobacteria (also called blue-green algae) or the toxic dinoflagellate blooms (e.g., *Karenia brevis*), the qualitative observation is effective to provide its approximate location and extent [88,89]. However, for blooms that are difficult to distinguish, even though some false positives will be generated, visual interpretation is still necessary as a preliminary procedure to avoid missing any initial symbols of a possible HAB event. If this step works successfully to provide preliminary information of a potential HAB event, further analysis is needed to extract the species composition and cellular optical characteristics for a full understanding of the target bloom [90].

### 4.2. Spectral Analysis and Classification

Compared to discoloration interpretation, spectral analysis of distinctive characteristics for HABs is more reliable at identifying monospecific blooms and extracting helpful information. Broadly, spectral characteristics involve two optical types: Apparent Optical Properties (AOP) and Internal Optical Properties (IOP). AOP is determined by the ambient light field and IOP is the absolute property of the medium itself [91]. Unique remote sensing reflectance (a typical AOP) patterns of HABs are determined by phytoplankton and have a close relationship with discoloration. Different classification algorithms or HAB identification rules are developed based on reflectance characteristics to discriminate HABs from other optical water types [3,92–94]. On the other hand, IOP such as absorption and backscatter are also widely examined to create optical models with the purpose of accurate identification of HABs [3,65,95]. Although influence by other components can contribute to the spectral shape of HAB waters, this step in the synthesized framework can still provide possible optical features of HABs and show the water information, such as the distribution pattern of algal bloom water, boundary of algal bloom water, eutrophication water, open ocean water and suspended sediment water [96].

### 4.3. Ocean Color Analysis

This step focuses on investigating corresponding oceanographic parameters obtained by ocean color techniques when monitoring a HAB event. The primary ocean color parameters, such as Chl-a and NPP, both of which are related closely to HAB events, are known to be biochemically informative. Therefore, tracing the spatial and temporal variation of such ocean color factors enables us to discover the presence of a changing HAB, especially large-geographic HABs lasting for a considerable period. High Chl-a and NPP zones in continuous sequential daily-images are promising indicators of the occurrence and movement of HABs [81]. In this framework, besides Chl-a, we particularly emphasize the analysis of NPP using remote sensing to explore its advantages in marine ecosystem study. Finally, the retrieval and spatial-temporal analysis of aforementioned ocean color parameters are expected to provide a substantive explanation from the biochemical perspective, leading to a more deep understanding of the true HAB mechanism.

### 4.4. Physical Oceanography Analyses

The fourth step of the proposed framework is to reveal a physical mechanism of HABs through examining physical oceanography variables such as SST and seawind. The same remote sensing approaches in ocean color analysis can also uncover the physical reasons for a large-scale HAB. SST retrieved by optical satellite data has advantages in detecting upwelling zones and water masses beneficial for HAB species propagation while seawind data acquired by microwave satellites is of great assistance in indicating the driving force of nutrients transportation and phytoplankton species accumulation. Winds shifts can be helpful in determining favorable and unfavorable conditions for the dynamic process of a HAB. The associated current and salinity data of study areas would offer additional information on physical contributors of some blooms [79,96,97]. Analysis carried out in this step is believed to strengthen the explanation of HABs from physical oceanographic perspectives.

### 4.5. Geological Explanations

This final step is to investigate the impacts of ocean topography on the formation and movement of the different types of currents which are related to HABs. For example, the steep slope and intensively changing topography underneath the Yangtze River Estuary and the adjacent East China Sea allows for the formation of coastal upwelling currents that promotes frequent HABs [59,98,99]. Another geological perspective is that of deposition characteristics. The deposition velocity can affect the formation of HABs because high velocity deposition can provide plenty organic nutrition for HAB species and is helpful for algae sporangiocyst bedding [59,100,101]. However, to obtain such geological information is beyond the capabilities of remote sensing techniques, but can be accessible by traditional geological and geophysical approaches such as seismic prospecting, magnetotelluric and multi-beam echo sounding techniques.

This present framework for the remote sensing of HABs integrates basic remote sensing approaches into one systematic flowchart to cope with the aforementioned problems. First, it provides a relatively full-scale view of the complicated mechanism of HABs allowing for a potential success in monitoring such marine phenomena. Besides, strategies applied in this framework can fully exploit the utilization of remote sensing techniques to a broader extent by involving a combination of optical and microwave satellite data, and considering sequential imagery of optimal resolutions for spatial–temporal analysis of parameters. Last but not least, this framework is intended to give multiple explanations of the complicated HAB mechanism from the different perspectives of biochemistry, physical oceanography and geology. Knowledge can also be enhanced regarding the spatial and temporal variation pattern of multiple oceanographic conditions during a HAB. This framework is meant to be an open system which can be improved by adding more effective modules for HAB detection if advanced remote sensing techniques emerge.

## 5. Conclusions

Remote sensing plays a significant role in investigating HABs over large geographical areas, although limitations still exist in current applications. There is no doubt that an effective monitoring and detecting system for remote sensing of HABs requires the coupling of powerful strategies and a comprehensive understanding of HAB oceanographic mechanisms. The synthesized framework proposed by this study allows for a potential method to present a systematical view of HABs, to improve the exploration of remote sensing techniques in HABs and to explain the complex nature of HAB mechanism from different oceanographic aspects. For progress to be most effective, future efforts should also be devoted to the fields of oceanographic and optical sampling, numeric modeling and the development of remote sensing algorithms. More accessible data and methods can be integrated into the established framework for combined analysis, to help address the current problems in improving routine-monitoring and forecasting of HABs.

## Figures and Tables

**Table 1.** Characteristics of historical and current ocean-color sensors (International Ocean Color Coordinating Group. http: http://www.ioccg.org/sensors).

**Sensor**	**Agency**	**Satellite**	**Operating Dates**	**Spatial Resolution (m)**	**Bands**	**Spectral Coverage (nm)**	**Obit**
**CZCS**	NASA (USA)	Nimbus-7 (USA)	24/10/78–22/06/86	825	6	433–12,500	Polar
**SeaWiFS**	NASA (USA)	OrbView-2 (USA)	01/08/97–14/02/11	1,100	8	402–885	Polar
**MODIS-Terra**	NASA (USA)	Terra (EOS-AM1)	Launch 18/12/99	250/500/1,000	36	405–14,385	Polar
**MODIS-Aqua**	NASA (USA) Ma'an	Aqua (EOS-PM1)	Launch 04/05/02	250/500/1,000	36	405–14,385	Polar
**Polder**	CNES (France)	ADEOS (Japan)	17/8/96–29/6/97	6,000	9	443–910	Polar
**Polder-2**	CNES (France)	ADEOS-II (Japan)	14/12/02–24/10/03	6,000	9	443–910	Polar
**Polder-3**	CNES (France)	Parasol	Launch 08/12/04	6,000	9	443–1,020	Polar
**MOS**	DLR (Germany)	IRS P3 (India)	21/03/96–31/05/04	500	18	408–1,600	Polar
**MERIS**	ESA (Europe)	ENVISAT (Europe)	Launch 01/03/02	300/1,200	15	412–1,050	Polar
**OCTS**	NASDA (Japan)	ADEOS (Japan)	17/08/96–29/06/97	700	12	402–12,500	Polar
**GLI**	NASDA (Japan)	ADEOS-II (Japan)	14/12/02–24/10/03	250/1,000	36	375–12,500	Polar
**OCI**	NEC (Japan)	ROCSAT-1 (Taiwan)	27/01/99–16/6/04	825	6	433–12,500	Polar
**OSMI**	KARI (Korea)	KOMPSAT-1/Arirang-1 (Korea)	20/12/99–31/1/08	850	6	400–900	Polar
**GOCI**	KARI/KORDI (South Korea)	COMS	Launch 26/6/10	500	8	400–865	Geostationary
**CMODIS**	CNSA (China)	SZ-3 (China)	25/03/02–15/09/02	400	34	403–12,500	Polar
**CZI**	CNSA (China)	Hy-1A (China)	15/05/02–01/04/04	250	4	420–890	Polar
**COCTS**	CNSA (China)	Hy-1A (China)	15/05/02–01/04/04	1,100	10	402–12,500	Polar
**CZI**	CNSA (China)	Hy-1B (China)	Launch 11/04/07	250	4	433–695	Polar
**COCTS**	CNSA (China)	Hy-1B (China)	Launch 11/04/07	1,100	10	402–12,500	Polar
**OCM**	ISRO (India)	IRS-P4 (India)	Launch 26/05/99	360/4,000	8	402–885	Polar
**OCM-2**	ISRO (India)	Oceansat-2 (India)	Launch 23/09/09	360/4,000	8	400–900	Polar
**MMRS**	CONAE (Argentina)	SAC-C (Argentina)	21/11/00 ∼ 2009	175	5	480–1,700	Polar
**HICO**	ONR and DODSpace Test Programme	JEM-EFInt.Space Stn.	Launch 18/09/09	100	124	380–1,000	51.6°,15.8 orbits p/d

## Appendix

**Table A1.** Studies on remote sensing of HABs focusing on different aspects.

**Chlorophyll**	**Chlorophyll & SST**	**Chlorophyll & Primary Production**	**Chlorophyll, SST, & Seawind**	**HAB indices or classification**
Balch *et al.* (1989) [62]; Stumpf *et al.* (2003) [20]; Gower *et al.* (2003) [45]; Gower *et al.* (2004) [46]; Hu *et al.* (2005) [42]; Reinart & Kuster [40]; Tomlinson *et al.* (2009) [43]; Zhao *et al.* (2010) [34];	Tang *et al.* (1999) [37]; Raine *et al.* (2001) [30]; Chang *et al.* (2003) [17]; Tang *et al.* (2003) [10]; Stumpf &Tomlinson (2005) [60]; Ahn *et al.* (2006) [11]; Tang *et al.* (2006) [41]; Sarangi & Mohammed (2011) [52]	Vargo *et al.* (1987) [23]	Tang *et al.* (2002) [15]; Tang *et al.* (2004) [5]	Haddad (1982) [93]; Prangsma &Roozekrans (1989) [71]; Danaher & Omongain (1992) [92]; Mao & Huang (1998) [72]; Gu *et al.* (1998) [73]; Kahru *et al.* (2000) [19]; Pasterkamp (2002) [94]; Ahn & Shanmugam (2006) [3]; Duan *et al.* (2008) [70]; Shen *et al.* (2011) [96]
